# The provision of written information and its effect on levels of pain and anxiety during electrodiagnostic studies: A randomised controlled trial

**DOI:** 10.1371/journal.pone.0196917

**Published:** 2018-05-14

**Authors:** Yan Ling Lai, Annemarie Van Heuven, Adeniyi Borire, Tejaswi Kandula, James G. Colebatch, Arun V. Krishnan, William Huynh

**Affiliations:** 1 Prince of Wales Clinical School, University of New South Wales, Sydney, New South Wales, Australia; 2 Institute of Neurological Sciences, Prince of Wales Hospital, Sydney, New South Wales, Australia; University of Würzburg, GERMANY

## Abstract

**Objective:**

The provision of written information is a low-cost and readily available intervention that has been found to reduce pain and anxiety in a variety of clinical settings. The current study was undertaken to determine if information provision may improve patients’ experience during conventional electrodiagnostic studies.

**Methods:**

128 participants were recruited from a tertiary teaching hospital who were referred for electrodiagnostic studies. They were randomized into 2 groups where the intervention group was provided with written information about the electrodiagnostic testing. Patients were invited to complete a questionnaire that included pain and anxiety using a visual analogue scale (VAS) following the testing. All participants underwent nerve conduction studies (NCS) whilst a subset also underwent subsequent needle electromyography (EMG).

**Results:**

Those who received information had a statistically significant lower perception of anxiety during NCS, whilst only females who received information had a statistically significant lower perception of pain to both NCS and EMG.

**Conclusions:**

The provision of written information can reduce the degree of pain and anxiety experienced during electrodiagnostic testing.

**Significance:**

Improving patient comfort and tolerability during electrodiagnostic testing may have practical implications towards more reliable and accurate results obtained from such investigations that may in turn improve patient diagnosis and management.

## Introduction

Due to the nature of electrophysiological evaluations, most patients express a degree of discomfort, pain and anxiety, with pain being the most common complication. This is well tolerated by most patients, however in some patients, poor tolerance may limit the validity and accuracy of the test by restricting the extent of the study and hence the diagnostic utility of these evaluations [1, 2]. This is particularly pertinent in the context of the ultimate value of these tests when taking into account the outcomes relative to the cost of the investigation. For a number of neuromuscular conditions, electrodiagnostic testing has been proven to have a dramatic impact on diagnosis and therapy [3].

Various interventions have been studied to increase tolerability of electrodiagnostic studies, particularly for pain related to needle electromyography (EMG). These include pharmacological interventions such as vapocoolant spray, eutectic mixture of local anaesthetics [4], ibuprofen [5] and paracetamol/tramadol [6]. Non-pharmacological interventions studied include listening to music [7], the provision of information [8], skin slapping [9], minimal insertion technique [10] to mention a few. The efficacy and safety of these have been well summarized in a recent article by London et al (2016) but the number of patients examined in these studies was small and none utilized a randomized study design.

Providing information that includes both sensory (sensations that will be experienced) and procedural (the sequence of events) aspects of the procedure a patient will be undergoing has been found to be beneficial in reducing stress and pain [11–13] in a number of surgical settings. These findings have been shown in recent studies of pre-operative patients [14], hip replacement [15], oral surgery [16] and preoperative anesthesia [17] and postoperatively as part of a multimodal intervention for managing pain [18]. In the setting of electrodiagnostic testing, conflicting results have been found. One study [19] found that it had no important effect whilst an another older study found that it decreased pain perception during nerve conduction studies (NCS) but not EMG [8]. Furthermore, there have also been reports of differences in experience with electrophysiological testing amongst genders [20, 21]. Given this discrepancy, the current prospective study sought to investigate, using a randomized control design, whether written information given to a larger cohort of patients undergoing electrodiagnostic studies for the first time, is an effective means of reducing their subsequent perceived pain and anxiety levels. Differences in experience amongst male and female patients were also further explored.

## Materials and methods

### Participants

This prospective study recruited patients whom were referred for outpatient NCS/EMG testing at a tertiary teaching hospital from October 2015 to December 2016. Participants were referred to the electrophysiology clinic for a wide range of provisional clinical diagnoses. These included most commonly entrapment neuropathies such as carpal tunnel syndrome, ulnar or peroneal neuropathy; peripheral neuropathy; radiculopathies; motor neuropathies or neuronopathies; and myopathy.

Inclusion criteria were: age between 18–80 years inclusive, English literacy (sufficient for comprehension of information pamphlet and questionnaire), willingness to participate in the study, no active psychiatric illnesses or cognitive decline, and not taking anti-neuropathic agents or psychotropic drugs.

Patients who met the above criteria were randomized on the day of testing by using a table of generated random numbers adapted from Pocock [22], where those assigned to even numbers received information whilst in the waiting room on the day of the study and those with odd numbers did not. Subjects were consented for the research study after NCS/EMG was completed.

**Fig 1:**
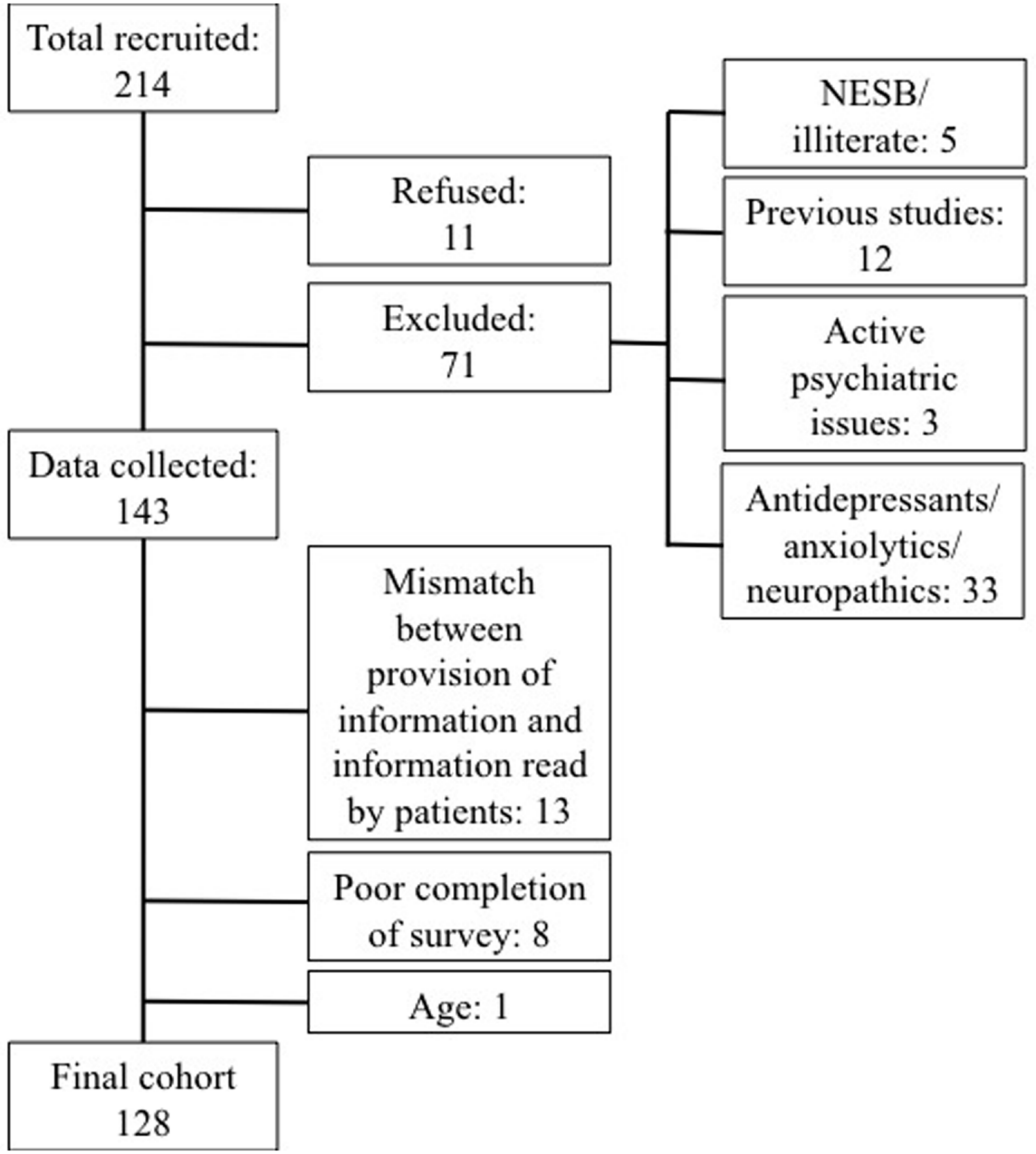
Flowchart of inclusion and exclusion criteria to achieve final cohort.

A total of 214 patients were initially recruited for the study over a one year period. Subsequent exclusions during data analysis were made based on the above-mentioned inclusion criteria or the questionnaire not being completed correctly. A total of 128 participants were included in the final data analysis and this was reached by further exclusion criteria (Fig 1). Amongst these, those participants who had previous NCS or EMG testing were excluded from the final cohort. In the final cohort, patients in the control group did not receive any self-reported additional information sources apart from basic verbal information provided by the examiner prior to the test. Patients in the intervention group would have received the information via the information brochure with or without further external information.

### Written information

Information was provided in the form of a 2 sided pamphlet informing patients about what to expect for the nerve conduction study, answering basic questions such as what the study would consist of, what patients needed to do during the test, what the test would feel like, how patients could prepare for the test, what to do if their symptoms were to get better before the test, how long the testing would take and whether it was safe if they had a pacemaker. On the second page, patients were informed that needle EMG may be performed subsequently and similarly, answered questions such as what it was, how many muscles would need to be tested, how they could prepare for it, what it would feel like, what they should expect after testing and when results would be received (S1 File).

### Blinding procedures, electrodiagnostic examination and questionnaire

Electrodiagnostic examination was performed by skilled examiners guided by senior clinicians. Examiners who performed the study were blinded to the assignment of information. Examiners provided a standard introduction and brief explanation regarding the testing procedure to all patients prior to the commencement of the electrophysiological testing. In addition, all patients were also given the opportunity to ask questions regarding the testing procedure.

All patients underwent NCS with or without subsequent EMG depending on the clinical indication. Electrical stimulation required for the NCS was standardised with the minimum stimulus required for an adequate examination as with typical studies. Immediately after the examination, patients who were eligible for the study completed a 2-page questionnaire in which they indicated their level of pain and anxiety experienced using separate 100 mm lines representing pain experienced during NCS, anxiety experienced during NCS, pain experienced during EMG and anxiety experienced during EMG, if applicable. The 100 mm line represented the visual analogue scale (VAS) scale (where 0 = no pain/anxiety on one end and 100 = worst pain/anxiety that they have ever experienced). The VAS scale, which is used as the primary outcome measure in this study, has been validated as a measure of the magnitude of experimental pain and anxiety [23–27].

The questionnaire included a secondary outcome measure of the experience of undergoing the study in relation to their expectation of the study. Further questions were whether they received information or had obtained information from other sources, whether they would have preferred to have received information, and the level of satisfaction with the electrodiagnostic examination. (S2 File).

The study received approval from the New South Wales Health South Eastern Sydney Local Health District Human Research Ethics Committee and written consent was obtained from all participating individuals.

### Statistical analyses

Data analysis was performed using IBM SPSS 23 statistical package. The Student's t-test was used when the distribution of data in both intervention and non-intervention groups were normally distributed, while the Mann-Whitney U test was performed when the data groups were non-normally distributed. The data is expressed as mean ± Standard Error of the Mean (SEM) and the p-value cut off of less than 0.05 was considered statistically significant. The planned comparisons between the gender subgroups also utilized the Student’s t-test or Mann-Whitney U test where appropriate. The Pearson chi-square test was used to study the differences in participant characteristics such as gender. It was also used to study the categorical data indicating patients’ experience after undergoing the test in relation to their expectation in the intervention group compared to the non-intervention group. Correlation analyses were performed using Spearman rank test.

## Results

Table 1 shows the baseline characteristics of participants in the NCS and EMG groups. There were no differences in age in the overall cohort (p = 0.13) as well as the EMG subgroup (p = 0.986), gender for the entire cohort (p = 0.22) and the EMG subgroup (p = 0.81), number of nerves studied (p = 0.88) and number of muscles (p = 0.08) sampled for both the intervention and non-intervention groups.

**Table 1:** Baseline characteristics of study population.

	Information received	No information received	p-value
**NCS**			
**N = 128**	64	64	
**Age**	53.34±2.05	49.22±1.75	p = 0.13
**Gender (F/M)**			p = 0.22
*Male*	33	26	
*Female*	31	38	
**Total NCS nerve studies**	8.38±0.36	8.23±0.35	p = 0.88
**Subgroup of patients who had subsequent EMG**			
**N = 28**	15	13	
**Age**	43.27±5.43	43.14±3.65	p = 0.99
**Gender (F/M)**			p = 0.81
*Male*	11	9	
*Female*	4	4	
**Total muscles sampled**	4.40±0.76	2.77±0.38	p = 0.08

### Primary outcomes

**Fig 2:**
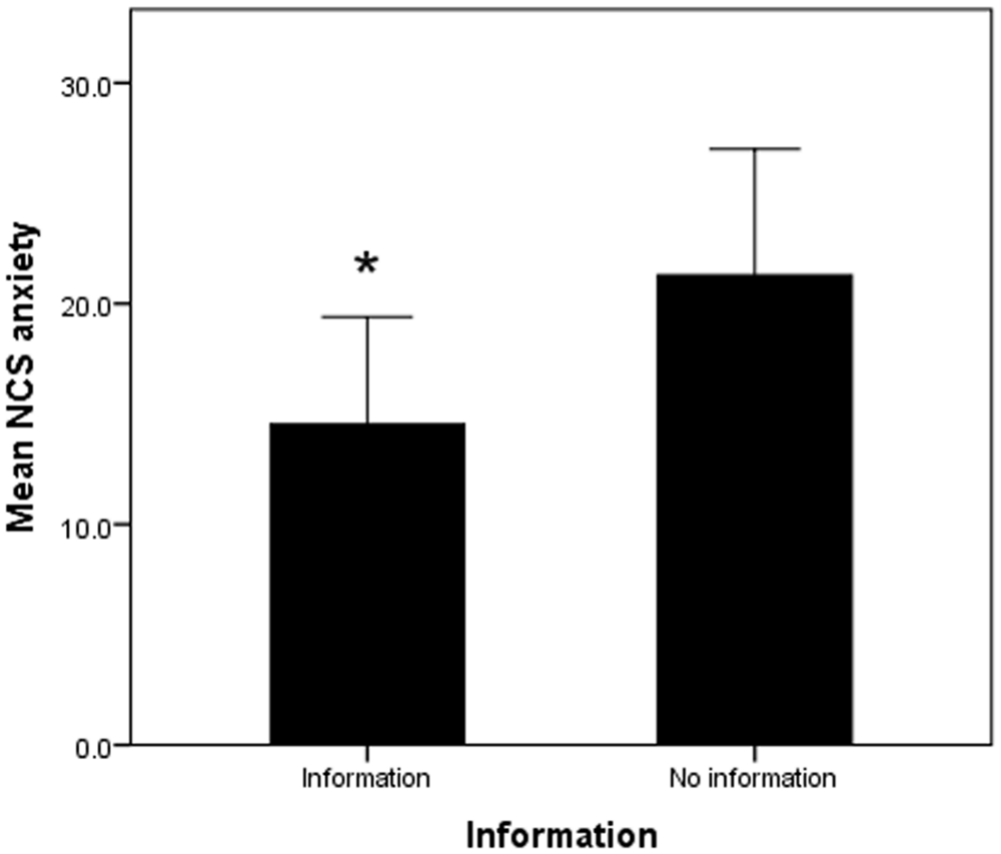
There was a significant difference observed in the effect of information provision on the level of anxiety perceived during NCS compared to those that were not provided with information.

Overall scores for anxiety in the NCS cohort were found to be significantly lower (p = 0.04) in the intervention group (14.53±2.44mm) than in the non-intervention group (21.28±2.86mm; Fig 2). Further subgroup analysis did not find a significant information effect by gender although in females, there was a trend when comparing those who received information (14.68±3.64mm) to those that did not (25.15±3.93mm, p = 0.052).

**Fig 3:**
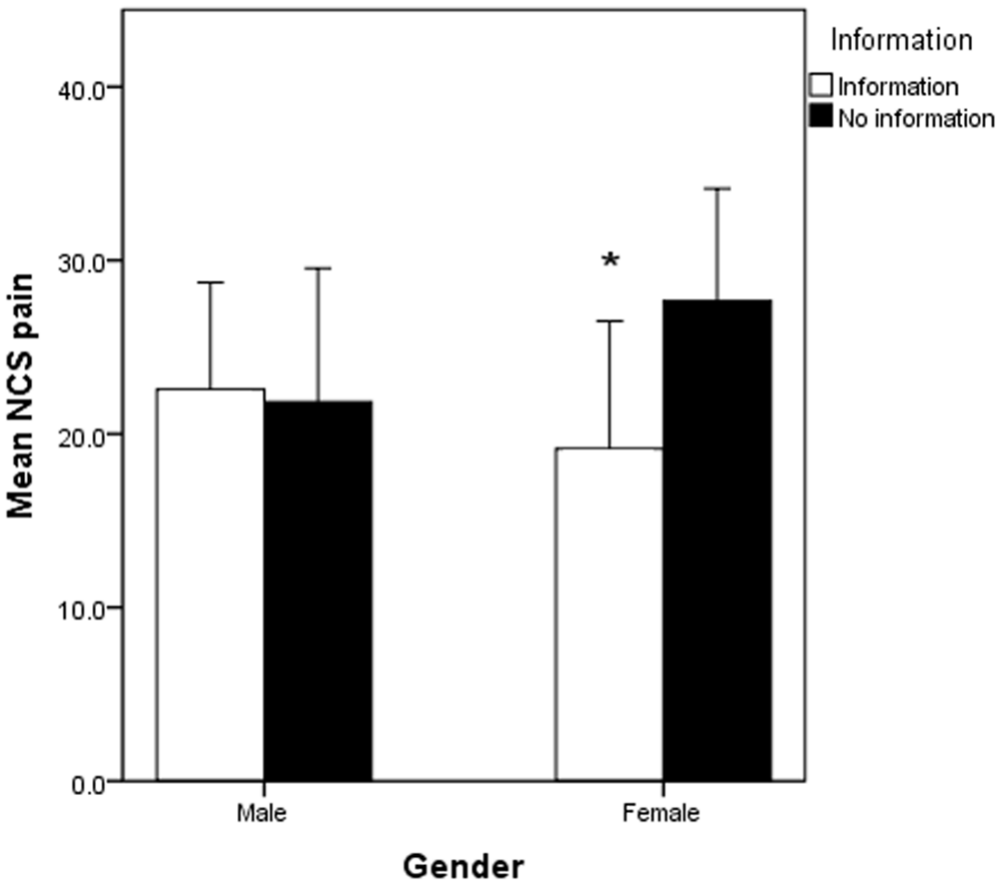
There was a significant difference observed in the effect of information provision on pain perceived during NCS in females but not in males.

Overall pain scores between the intervention (20.91±2.33mm) and non-intervention group (25.30±2.44mm) in the NCS cohort did not differ significantly (p = 0.15). Further gender subgroup analysis was performed but there were no significant differences in pain scores amongst males in the intervention group (22.56±3.03mm), compared to those in the non-intervention group (21.85±3.74mm; p = 0.71). However, amongst females, NCS pain scores were found to be significantly lower (p = 0.033) in the intervention group (19.15±3.60mm) compared to the non-intervention group (27.66±3.19mm; Fig 3).

**Fig 4:**
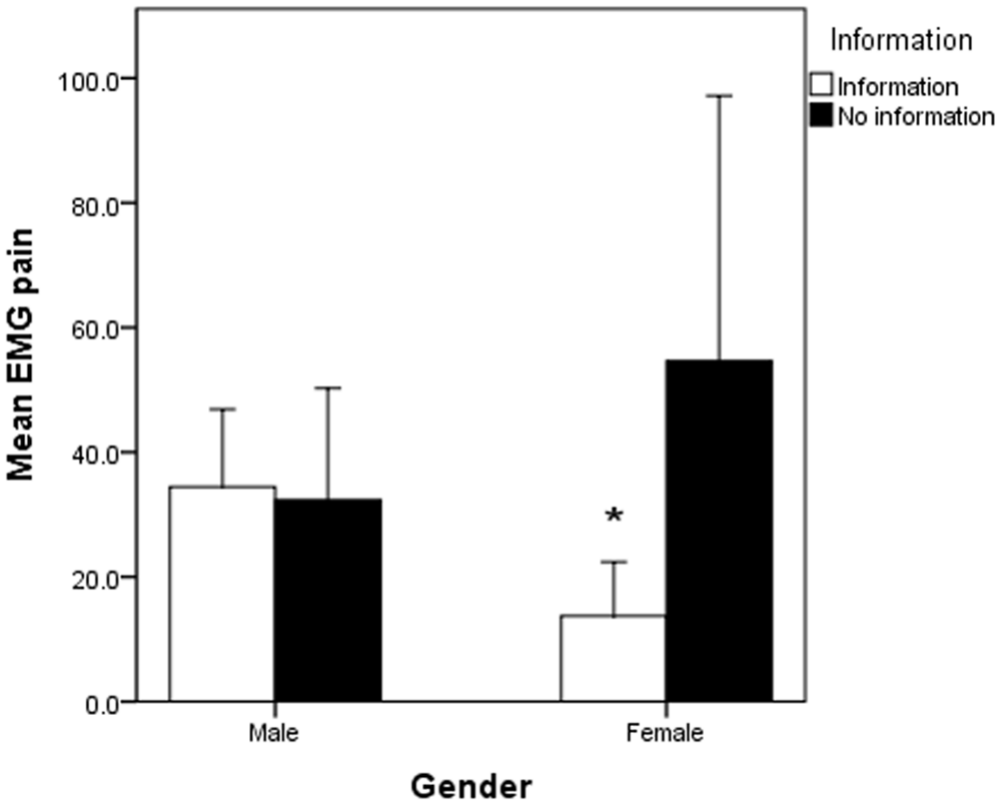
There was a significant difference observed in the effect of information provision on pain perceived during needle EMG testing in females but not in males.

**Fig 5:**
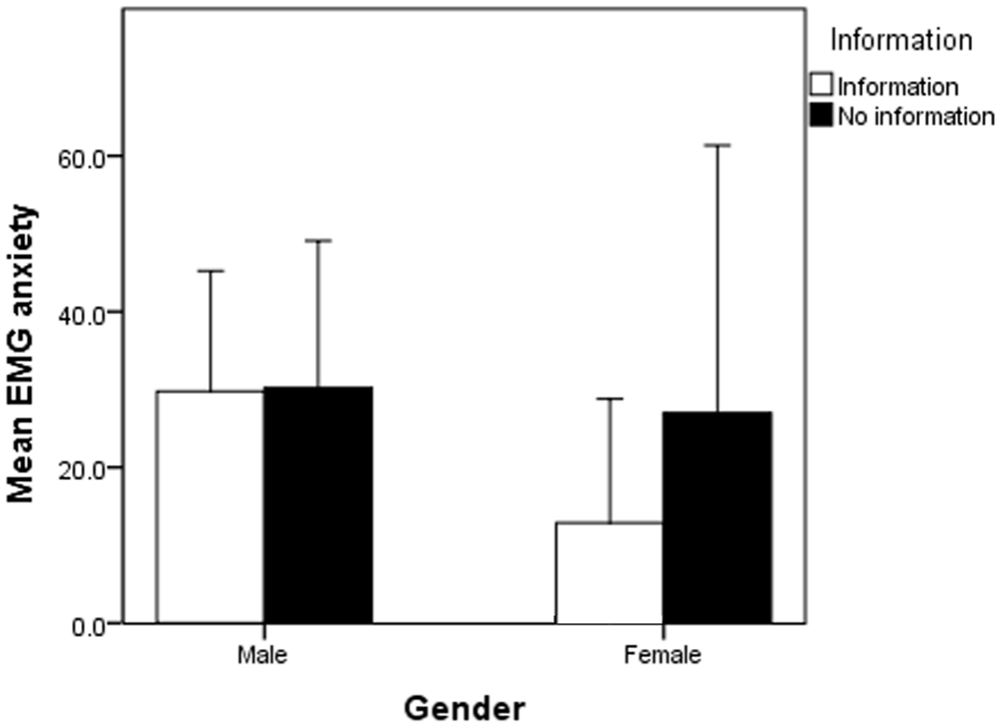
There were no significant differences observed in the effect of information provision on the level of anxiety perceived during needle EMG testing.

No significant differences in the perceived pain (p = 0.34) or anxiety (p = 0.65) levels were demonstrated for the intervention and non-intervention groups in the subgroup of patients that underwent needle EMG testing. However, when further subgroup analysis by gender was performed, a significant information effect (p = 0.024) was found in females in the level of pain perceived during the EMG testing. Those who received information had significantly lower levels of pain (13.75±2.71mm) compared to those that did not (54.63±13.37mm) (Figs 4 and 5).

### Secondary outcome measure

Amongst patients who received information compared to those who did not, those who received information showed a trend in reporting the study experience as being better than expected rather worse or same as expected (p = 0.055). Furthermore, females were significantly more likely to want the provision of information than men (p = 0.014).

## Discussion

This is the first study utilizing a randomized control design in a large cohort of patients examining the effect of the provision of written information on the perceived levels of pain and anxiety during conventional electrodiagnostic testing. The use of VAS in retrospective pain assessments have been found to accurately reflect patients' pain experience during the procedure [28] and up to 3 months [29]. Concurrence in retrospective assessments in anxiety has also been similarly demonstrated [30].

The results of the current study demonstrated that compared with patients who were not provided with information, those who were provided with information showed a significant reduction in the anxiety levels perceived during NCS. It is interesting to note that for patients who received information compared to those who did not, there was a trend of reporting the study experience as being better than expected rather than worse or the same as expected. This is in keeping with previously reported data demonstrating that the amount of presented information allowed an increase in the perception of patient control that ultimately assisted in the management of acute procedural pain [31–33].

In females a reduction in pain levels was previously observed during NCS and EMG testing [20, 21]. While anxiety has been found to influence the level of pain experienced [34, 35], subgroup analyses assessing the effect of information on experienced levels of anxiety between genders did not reveal a statistically significant difference. This suggests that the reduction of pain experienced amongst females in our current study would not be adequately explained by a reduction in anxiety alone. Regarding information and cognitive control as it relates to the degree of experienced pain [36], it has been suggested that females have greater interest and concern regarding health matters [37]. As such, females in the current study who were not provided with prior written information, were significantly more likely to indicate that they would have preferred to have been given such information. Such an effect was not seen in males.

The reduction in EMG pain perceived in females who received information compared to their male counterparts is consistent with the suggestion that there are gender differences in responses to non-pharmacological interventions such as cognitive intervention [38] for experimental pain [39]. Additionally, it has been suggested that predictors of effective treatment may be influenced by gender [39].

A much smaller sample of patients was collected for needle EMG compared to NCS and may represent a potential limitation of the study. The relative number of patients who went on to have EMG was small in our cohort as many of the referrals were for CTS, ulnar neuropathies, peripheral neuropathies, which in most clinical circumstances would not require needle EMG subsequent to the diagnostic NCS. Study group numbers for comparing pain in females during EMG was also small, however, it should be noted that means of groups were reflective of group perceptions. Future studies that are able to recruit larger numbers for needle EMG patients may be of benefit to corroborate the current findings.

In conclusion, the provision of written information as a simple intervention to reduce subsequent pain and anxiety experienced during electrodiagnostic studies is effective, practical and easily implemented. Prior information is particularly effective in improving the tolerability of the study for females. This has important practical implications as improved tolerance to the testing may facilitate improved reliability and accuracy of electrodiagnostic results as well as allowing for more thorough testing which may in turn result in improved diagnosis and management for patients.

## Supporting information

S1 File Written information. Those participants whom were randomized to received written information were provided with this in print.
(PDF)

S2 File Sample questionnaire. All participants whether provided with written information or now prior to the electrophysiological testing, were asked to complete the study questionnaire if they consented to take part in the study.
(PDF)

## Abbreviations

EMG: Electromyography
NCS: Nerve Conduction Study
SD: Standard deviation
SEM: Standard Error of the Mean
VAS: Visual Analogue Scale
