# Supplementary material for: The provision of written information and its effect on levels of pain and anxiety during electrodiagnostic studies: A randomised controlled trial
Source: PLoS One. 2018 May 14;13(5):e0196917. doi: 10.1371/journal.pone.0196917 (PMC5951568; doi:10.1371/journal.pone.0196917)
Supplement: S1 File — Those participants whom were randomized to received written information were provided with this in print. (PDF) [file pone.0196917.s001.pdf]

Nerve Conduction Studies (NCS) and Electromyography (EMG): What to expect

Your doctor has referred you for these tests as a way of assessing the health of your nerves and/or muscles. There are several different testing procedures, but the basic testing is the same.

What is a nerve conduction study (NCS)?

‘This is an electrical test of your nerves. The doctor who performs this test will attach some wires and electrodes to your skin, usually on your hands or feet, and encourage you to relax the limb. Then they will give a brief series of electrical impulses through the wires, which will start at zero and gradually build up in strength until the best response is obtained from your nerve. A computer will measure how quickly your nerves conduct the electrical information and how much of the information gets through. This gives us an idea how well your nerve is functioning.

The tests will look at a variety of nerves, and will include tests of both sensation function of the nerves and also motor function of the nerves. The motor tests will make your muscles jump, but this is not painful – just a funny feeling!

What do I need to do during the test?

It is important that you let your muscles stay loose and relaxed during the testing, as this will help get the best possible results and will also minimise the number of electrical impulses that we need to give you.

You do not need to tell the doctor when you feel the impulses unless they ask you. Your job is to lie back and relax! We will instruct you on how to position yourself for the tests.

What do NCS feel like?

The impulses feel a bit like the static feeling that you might get when you touch a car door or shake hands with someone. They are not painful, but they are a bit uncomfortable. It is normal to feel a bit concerned before the test, but the vast majority of patients are able to get through the testing without any difficulty.
How do I prepare for the test?

To prepare for the test, please keep your arms and legs warm, but wear loose clothing so that your upper arms and thighs can be accessed if needed. Light layers are ideal. You can take all of your usual medication, but please inform the doctor if you are on anticoagulation (blood thinners).

What if my symptoms get better before the test?

It doesn’t matter if your symptoms aren’t there on the day of the test; the testing is still useful. Often we can still pick up abnormalities, and even if the testing is normal it provides a baseline measurement of your nerve responses to compare future tests to.

How long does it take?

This depends on the reason for the test. A simple study of the nerves in the hands might take twenty minutes, whereas a more complicated test of many nerves in the whole arm, including needle EMG testing, might take forty-five minutes to an hour. In general allow between 30-60 minutes for your test.

Certain specialized tests, such as single fibre EMG (SFEMG) may take an hour or longer, however usually we will inform you of this in advance.

I have a pacemaker, is it safe?
Yes, so long as the tests are not performed close to the pacemaker, nerve conduction studies are safe. Please let the doctor know that you have a pacemaker.

What is electromyography (EMG)?
EMG is an extra test that is sometimes performed in addition to the nerve conduction studies to give your doctor more information about your nerves and muscles. The doctor performing the test will decide whether it would be useful to perform EMG as well as NCS. It involves a very fine needle being inserted into a muscle, and then a computer measures the natural electrical activity of that muscle and displays that information as sound and pictures. We do not give you any electrical impulses during this test, we just listen to the electricity that your body naturally produces. It can get quite noisy, and sounds like an untuned radio, popcorn, or rain on a tin roof. We listen to your muscle first at rest and then will show you how to move that muscle so we can listen to it when it is contracting at full strength. The sounds and waveforms tell us whether the nerve supply to the muscle is healthy, and whether the muscle itself is normal.

How many muscles have to be tested?
This will depend on the reason for the test. It may be as few as one or two, or up to several muscles in each limb.

How do I prepare for this test?
Preparation is the same as for a nerve conduction study.

What does EMG feel like?
Because we use a needle, there is a small amount of pain associated with this test. However it should be minor as the needle we use is very thin (smaller than a blood test needle). If you are finding it painful, please let the doctor know as we may be close to a nerve ending and sometimes moving the needle will make it more comfortable for you.

What should I expect after the test?
Nerve conduction studies cause no side effects. After a needle EMG you may experience some mild tenderness or bruising in the tested muscles, this should settle within a few days. If there is bleeding, put pressure on the site until the bleeding stops and see a doctor if it persists.

When do I get my results?
We will send a copy of the results to your doctor within a few days of performing the test. If you would like an extra copy sent to another doctor (e.g. a specialist you are seeing or your GP) then please let our reception staff know.
