# Supplementary material for: The provision of written information and its effect on levels of pain and anxiety during electrodiagnostic studies: A randomised controlled trial
Source: PLoS One. 2018 May 14;13(5):e0196917. doi: 10.1371/journal.pone.0196917 (PMC5951568; doi:10.1371/journal.pone.0196917)
Supplement: S2 File — All participants whether provided with written information or now prior to the electrophysiological testing, were asked to complete the study questionnaire if they consented to take part in the study. (PDF) [file pone.0196917.s002.pdf]

Nerve Conduction studies (NCS) and electromyography (EMG)

Patient Questionnaire

Lab ID No………………………. **Date of testing…………………..**

1. Please indicate the level of ***pain*** experienced during the Nerve Conduction Study (**electric impulse test**) by marking a cross on the line below:

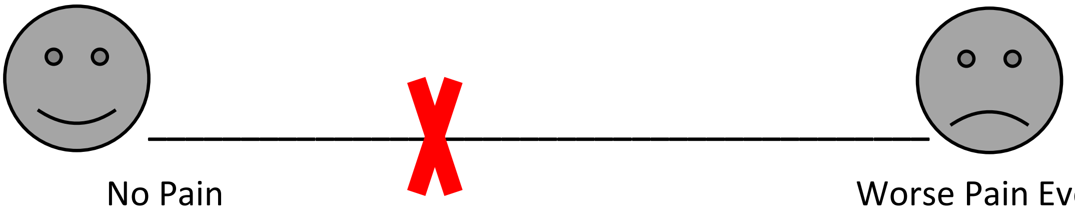

No Pain Worse Pain Ever

2. Please indicate the level of ***anxiety*** experienced during the Nerve Conduction Study (**electric impulse test**) by marking a cross on the line below:

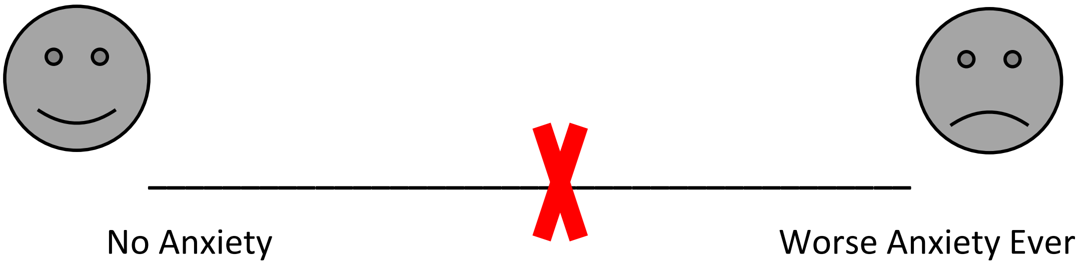

No Anxiety Worse Anxiety Ever

3. If you also had the **needle test** (EMG), please indicate the level of ***pain*** experienced during this:

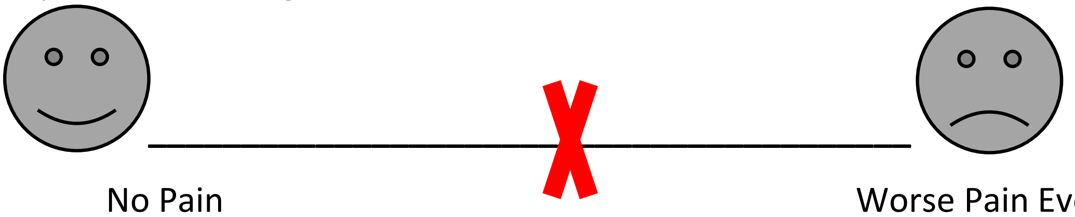

No Pain Worse Pain Ever

4. If you also had the **needle test** (EMG), please indicate the level of ***anxiety*** experienced during this:

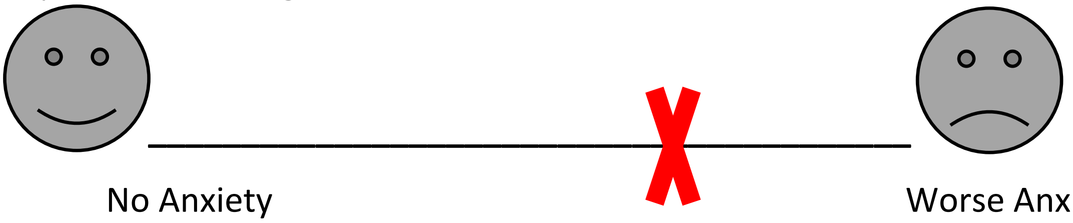

No Anxiety Worse Anxiety Ever

5. Was the testing today (tick one):

- ☒ Better than expected
- ☐ Worse than expected
- ☐ Same as expected

6. Were you provided with a printed handout with written information about the test today?

- ☐ Yes.
  - If so, did you read it? Yes / No
- ☒ No.
  - If so, did you read about the test elsewhere before coming today? Yes / No
  - Would you like to have been given information beforehand today? Yes / No

7. How satisfied overall were you with the testing today?

0 — 5 — 10

Not satisfied — Extremely satisfied

8. Have you had this sort of study in the past? Yes / No
